# Using ecological socioeconomic position (SEP) measures to deal with sample bias introduced by incomplete individual-level measures: inequalities in breast cancer stage at diagnosis as an example

**DOI:** 10.1186/s12889-019-7220-4

**Published:** 2019-07-02

**Authors:** Sébastien Lamy, Florence Molinié, Laetitia Daubisse-Marliac, Anne Cowppli-Bony, Stéphanie Ayrault-Piault, Evelyne Fournier, Anne-Sophie Woronoff, Cyrille Delpierre, Pascale Grosclaude

**Affiliations:** 1 Laboratory of Epidemiology and Analyses in Public Health, Faculté de Médecine, UMR 1027 Inserm – Université Toulouse 3 Paul Sabatier, Equipe EQUITY labellisée par le Ligue nationale contre le cancer, 37 allées Jules Guesde, F-31000 Toulouse, France; 2 French network of Cancer registries (Francim), F-31000 Toulouse, France; 3 Loire-Atlantique / Vendée Cancer Registry, F-44093 Nantes, France; 4 SIRIC ILIAD, Nantes University Hospital, F-44093 Nantes, France; 5 Tarn Cancer Registry, University Cancer Institute of Toulouse – Oncopole (IUCT-O), F-31000 Toulouse, France; 6 Doubs and Belfort territory Cancer Registry, Besançon University Hospital, F-25000 Besançon, France; 7 Resarch Unit EA3181, Universiy of Franche-Comté, F-25000 Besançon, France

**Keywords:** Social inequalities, Methodology, Cancer, Population-based data, Deprivation

## Abstract

**Background:**

When studying the influence of socioeconomic position (SEP) on health from data where individual-level SEP measures may be missing, ecological measures of SEP may prove helpful. In this paper, we illustrate the best use of ecological-level measures of SEP to deal with incomplete individual level data. To do this we have taken the example of a study examining the relationship between SEP and breast cancer (BC) stage at diagnosis.

**Methods:**

Using population based-registry data, all women over 18 years newly diagnosed with a primary BC in 2007 were included. We compared the association between advanced stage at diagnosis and individual SEP containing missing data with an ecological level SEP measure without missing data. We used three modelling strategies, 1/ based on patients with complete data for individual-SEP (*n* = 1218), or 2/ on all patients (*n* = 1644) using an ecological-level SEP as proxy for individual SEP and 3/ individual-SEP after imputation of missing data using an ecological-level SEP.

**Results:**

The results obtained from these models demonstrate that selection bias was introduced in the sample where only patients with complete individual SEP were included. This bias is redressed by using ecological-level SEP to impute missing data for individual SEP on all patients. Such a strategy helps to avoid an ecological bias due to the use of aggregated data to infer to individual level.

**Conclusion:**

When individual data are incomplete, we demonstrate the usefulness of an ecological index to assess and redress potential selection bias by using it to impute missing individual SEP.

**Electronic supplementary material:**

The online version of this article (10.1186/s12889-019-7220-4) contains supplementary material, which is available to authorized users.

## Background

Socio-economic position (SEP) is multidimensional and can affect health through different mechanisms across the life-course [1–3]. SEP can be measured by different ways [4, 5]. When SEP is addressed using direct measures at the individual level, it consists usually in collecting level of education, income, and occupation. However, these data are rarely collected routinely in medical administrative databases and cancer registries. Indeed, to access such data, it is needed to develop ad hoc surveys such as cohort studies. However, in such studies the proportion of missing data due to attrition may be high which can limit the usefulness of this kind of studies. To overcome this limitation, the use of ecological-level measures as a proxy of individual SEP is an alternative [6, 7]. This assumes that all individuals have the same SEP than those of the area to which they belong. Obviously, this is not true as area-level SEP-related data derived generally from summary statistics of individual’s characteristics, collected in routine (for instance in census surveys) and aggregated at an area-level because of privacy and personal data protection. Area-level measures of SEP translate the average SEP over all the people in the area. Thus, using such tools to measure the individual SEP is associated to measurement errors that may translate into a lack of accuracy associated with a loss of statistical power. This error is bigger when the area used to approximate individual SEP is large as individuals SEP in large areas are likely to be more heterogeneous than in smaller areas. Among area-level measures of SEP, ecological deprivation indexes offer an opportunity to apprehend several dimensions of SEP in one measure. Deprivation was introduced by Townsend as a state of observable and demonstrable disadvantage relative to the local community or the wider society to which an individual, family or group belongs [7]. Ecological deprivation indexes are now easily available. When the focus is on individual SEP, but these data are missing, deprivation index are used as a proxy of individual-level of SEP, despite a measurement error. When individual social data are available but incomplete due to missing data, the best way to uses deprivation indexes is not clear: they are often used in addition to additional individual data to characterize the context even if they are not designed to do such work.

In the present paper, we discuss an alternative use of ecological deprivation indexes where individual measures are collected with missing data, i.e. using ecological deprivation indexes to deal with missing data at the individual level. To illustrate this, we focus on the influence of SEP on breast cancer (BC) stage at diagnosis which is largely supported by the literature [8, 9], including in France [10]. These studies largely supported an overrepresentation of late-stage cancer at diagnosis among patients with low socioeconomic positions.

## Material and methods

### Data origin and description

The data presented came from three population-based cancer registries of the Francim network (Doubs, Loire-Atlantique, and Tarn). The study has been described in detail elsewhere [11]. Briefly, all women over 18 years old residing in the area covered by the registries and newly diagnosed with a primary BC in 2007 were included. Men, women with known antecedent of BC, lymphomas or sarcomas were not included. Women with no information regarding stage at diagnosis and whose residence address was not known were excluded. Data regarding demographical and clinical characteristics were collected directly by the cancer registries from clinical records in care centres. A self-administered questionnaire was sent to women to collect health behaviours, care trajectory before the first hospitalization and their individual socioeconomic position (SEP). Women were classified as respondents if they fully completed the self-administered questionnaire and as non-respondents if they did not complete the questionnaire or if they had a question on which no data regarding individual SEP were available. The study was approved in 2008 by national ethical committees (CNIL (n°907,172) and CCTIRS (n°08075)).

### Stage at diagnosis

It was defined in accordance with the 5th TNM (Tumor, Nodes, Metastasis) classification for malignant tumours [12]. We defined two groups regarding their initial prognosis: early stage BCs (Tis/T1 N0 M0) and advanced stage BCs (T2/T3/T4 N0 M0, or N+, or M+).

### Women’s socioeconomic position

#### Individual-level SEP measure

Three SEP variables were used to approach the individual social deprivation: the level of education (lower than high school, high school or higher), occupation (manual, non-manual), and income (tax exemption, or not). We chose to create a multidimensional individual index to capture broader dimensions of individual deprivation. Each SEP variable was scored 1 in case of lower socioeconomic environment (education lower than high school, manual worker, and tax exemption) or 0 otherwise. Those three variables were then summed to compute an individual-level deprivation index (IDI) ranged from 0 (least deprived) to 3 (most deprived).

#### Area-level SEP measure

Women’s address at diagnosis was collected and used to obtain the corresponding smallest geographical unit available at the time of the study in France for the statistical information ((IRIS) “Îlots Regroupés pour l’Information Statistique”) by geolocation. An IRIS represents in average 2000 inhabitants relatively homogenous in terms of socioeconomic characteristics. We used the 2007 French version of the European deprivation index (EDI) which is available at the IRIS level. It has been developed with the ambition of being transposed to other European countries [13]. To date EDI exists for France, Italy, Spain, Portugal and England [14]. Each IRIS was assigned an EDI value, calculated from the 2007 census data. A high value means an IRIS with a high level of deprivation. Quintiles from the national distribution of EDI were computed from all the IRIS of metropolitan France (excluding overseas regions), from quintile 1 corresponding to the least deprived to quintile 5 corresponding to the most deprived IRIS.

#### Individual-level SEP measure with missing data imputed

To impute missing values in IDI, we used the multiple imputations using chained equation procedure in STATA to generate 20 sets of plausible values of IDI based on an imputation model depending on EDI and covariates [15, 16]. The imputation model was constructed using variables assumed to be associated with IDI: age, health insurance regimens (regimens and supplementary universal healthcare coverage allocated under an income threshold giving entitlement, for patients with lower income), tumour characteristics (human epidermal growth factor receptor 2 (HER2), oestrogen and progesterone receptor status, Scarff-Bloom-Richardson (SBR) grade),living place (geographical area, rural/urban classification, and distance to the nearest gynaecologist), and EDI that has been showed as a relatively good predictor of individual deprivation [17]. According to Sterne et al. who recommend to include the outcome in the imputation model, stage at diagnosis was introduced in the imputation models [18]. It results in the imputed IDI (i-IDI) coded in the same way as IDI.

### Statistical analysis

We tested first (using Chi-square tests), the selection bias due to the exclusion of women with incomplete data on IDI. Indeed, compared to respondents, non-respondents might have more often poor SEP and advanced stage at diagnosis. Then we tested for the association between SEP and advanced stage at diagnosis under different configurations: i) Firstly we compared the results obtained using either IDI or EDI among women with complete data on SEP (model 1: complete case analysis). We assumed that due to the aggregated nature of EDI, the results using this index are exposed to measurement errors leading to a lack of accuracy and a loss of power in the assessment of individual SEP-related differences in stage at diagnosis; ii) Then we studied the association between SEP and advanced stage at diagnosis using EDI among women for whom individual measures of SEP were missing (model 2: missing case analysis) in order to approach what may be the effect on results if these women had been included in the analyses 1; iii) Next, we studied the association between SEP and advanced stage at diagnosis using i-IDI among respondents and non-respondents (model 3: imputed case analysis). We assumed that in this configuration the selection bias observed in complete case analysis would be downward as non-respondent women would be included. By increasing sample size compared to complete case analysis, we expected to observe stronger association between i-IDI and stage at diagnosis than what is observed using IDI; iv) And finally, we studied the association between SEP and advanced stage at diagnosis using EDI among women with data on either IDI or EDI (model 4: proxy measure analysis). As in imputed case analysis, we assumed that the selection bias observed when excluding women with missing individual SEP would be downward in this configuration. However, we assumed that the observed associations would be attenuated compared to results using i-IDI due to the loss of accuracy and power deriving from the use of an ecological index to approach the individual level. We focused on the crude association between SEP and stage at diagnosis and thus, we did not adjust our models for confounders including age. In sensitivity analyses, the models were adjusted for age. We also tested the influence of excluding EDI from the imputation model on the model 3 results to test for the contribution of EDI in the imputation. For each analysis, we used logit models with advanced stage at diagnosis as outcome. We used area under ROC curve (AUC) to compared models using different SEP measures in terms of classification errors. This was done using the test based on Delong’s et al. algorithms [19]. We used 0.2 and 0.05 as statistical significance levels for respectively the comparison between respondents and non-respondents and regression analyses. Statistical analyses were performed with STATA software version 14 (StataCorp LP, College Station, TX, USA).

## Results

**Fig. 1:**
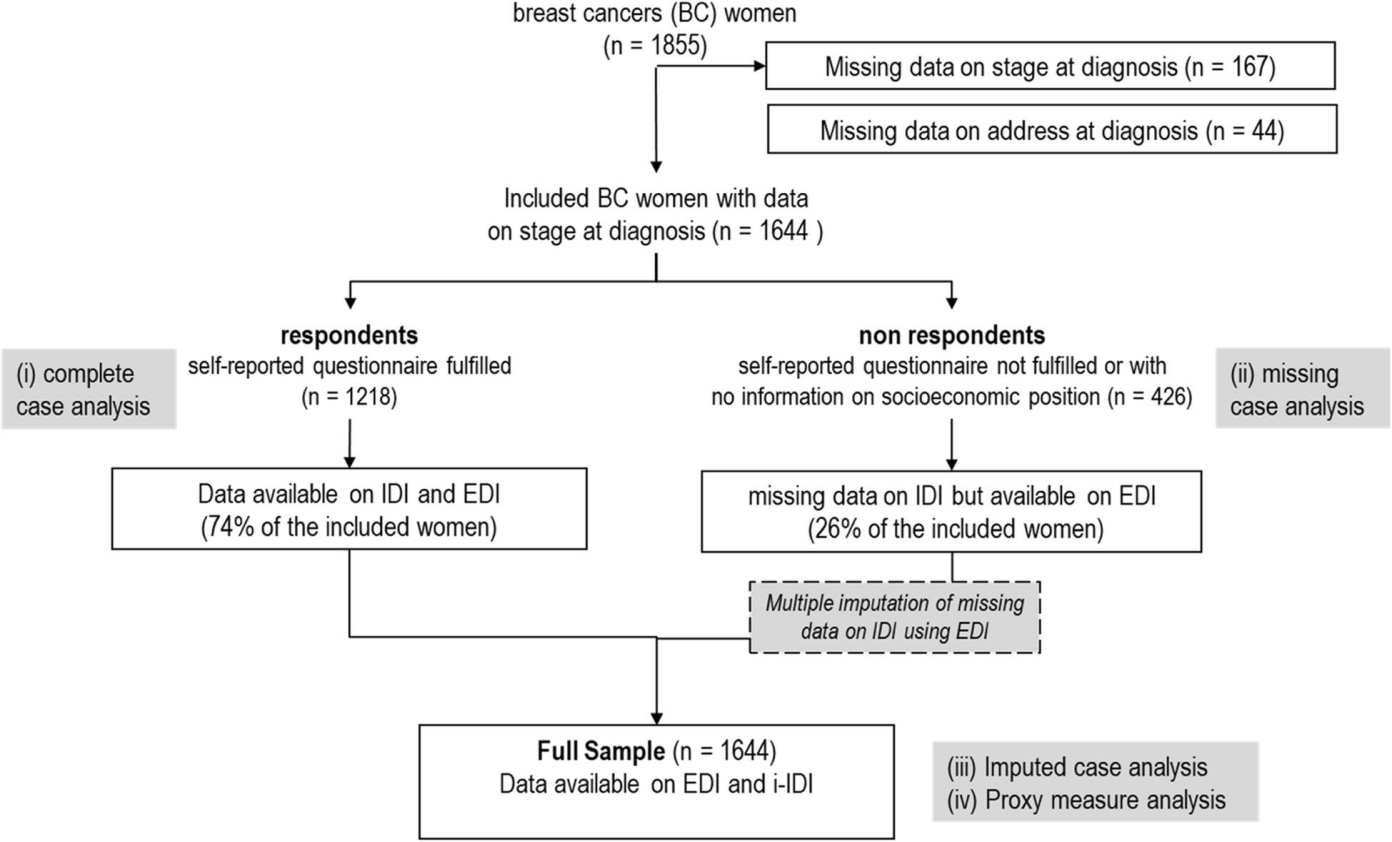
Flowchart

Initially, 1855 women identified as eligible to the study. About 89% of them were included, representing 1644 women with data regarding address and stage at diagnosis. Among those, 1218 respondents, i.e. women who responded to the self-reported questionnaire and had data on IDI and EDI. There were 426 non-respondents, i.e. women who did not response the self-reported questionnaire or did not fulfil the questions regarding SEP, and thus had no data regarding IDI. The flowchart is presented in Fig. 1.

The choice of the SEP measure influences the size of the sample to analyse. Choosing the individual-level SEP may also expose analyses to a selection bias due to the exclusion of the 426 women with no data on IDI, i.e. one in four women initially included. Indeed, Table 1 shows that, compared to women who have responded to questionnaire and have then data on IDI (*n* = 1218), those who did not respond were significantly older and with more advanced stage at diagnosis. The distribution of the respondents and non-respondents in the EDI quintiles was not statistically significant (*p* = 0.361). However, the comparison of the proportion of patients living in the highest EDI quintile (most deprived) differed statistically between the respondents and the non-respondents (chi2 test comparing EDI (q1-q4 vs q5) between groups, *p* = 0.067).

**Table 1:** Characteristics of included women depending on the availability of data on IDI

		respondents (IDI available (*n* = 1218))		non-respondents (missing data on IDI (*n* = 426))		
		N	*%*	N	*%*	*p*-value*
age	less than 50	278	*22.8*	88	*20.7*	<0.001
	50 to 74	798	*65.5*	231	*54.2*	
	75 or more	142	*11.7*	107	*25.1*	
stage at diagnosis	Early	708	*58.1*	205	*48.1*	<0.001
	advanced	510	*41.9*	221	*51.9*	
EDI	q1 (least deprived)	307	*25.2*	97	*22.8*	0.361
	q2	300	*24.6*	99	*23.2*	
	q3	265	*21.8*	89	*20.9*	
	q4	202	*16.6*	76	*17.8*	
	q5 (most deprived)	144	*11.8*	65	*15.3*	

*Chi-square test

In Table 2, in complete case analysis (model 1), results from the model with IDI showed a significant association between IDI and stage at diagnosis. We observed a higher risk of being diagnosed with advanced stage among women with the highest values of IDI, with a statistically significant IDI-related gradient. Contrarily, among the same sample, the use of EDI failed to observe similar results. None gradient nor association between one EDI level and stage at diagnosis were observed. Among the 1218 respondents, the proportion of advanced stage cancer from IDI score 0 to 3 were respectively 40, 41 44 and 52%. Conversely, these proportions from EDI quintile 1 to 5 were respectively 40, 43, 42, 43 and 41%.

**Table 2:** Complete cases analysis among the 1218 respondents. Model 1 addressing the crude association (Odds ratios [95% confidence intervals]) between SEP (either IDI or EDI) and stage at diagnosis (advanced vs early stage (ref.))

		OR	[95%CI]		*p*-value
model 1.a with individual-level SEP measure (*n* = 1218)					
Individual-level Deprivation Index (IDI)	0 (least deprived)	1			
	1	1.05	[0.79;	1.39]	0.737
	2	1.20	[0.87;	1.66]	0.262
	3 (most deprived)	1.62	[1.10;	2.40]	0.014
Area under the ROC curve (AUC) = 0.533					
model 1.b with area-level SEP measure (*n* = 1218)					
European Deprivation Index (EDI)	quintile 1 (least deprived)	1			
	quintile 2	1.16	[0.84;	1.61]	0.369
	quintile 3	1.11	[0.79;	1.55]	0.540
	quintile 4	1.15	[0.80;	1.65]	0.455
	quintile5 (most deprived)	1.05	[0.70;	1.57]	0.803

AUC = 0.516

In missing case analysis (model 2), Table 3 shows a strong association between stage at diagnosis and SEP using EDI, with an EDI-related gradient. This illustrates the selection bias that occurs when women who did not respond to the self-reported questionnaire are excluded. This bias may likely lead to an underestimation of the SEP-stage at diagnosis relationship when these women are excluded from the analysis. The non-respondent women are older, with more advanced stage at diagnosis and tended to be more deprived than the respondent. In this sub-population, the proportion of advanced stage cancer from EDI quintile 1 to 5 were respectively 37, 52, 51, 61 and 66% (*p*-value< 0.01; Chi-square test with 4 df, data not shown).

**Table 3:** Missing cases analysis among the 426 non-respondents. Model 2 addressing the crude association (Odds ratios [95% confidence intervals]) between SEP (EDI) and stage at diagnosis (advanced vs early stage (ref.))

	*N* = 426	OR	[95%CI]		*p*-value
European Deprivation Index (EDI)	quintile 1 (least deprived)	1			
	quintile 2	1.80	[1.02;	3.20]	0.043
	quintile 3	1.73	[0.97;	3.11]	0.066
	quintile 4	2.60	[1.40;	4.82]	0.002
	quintile 5 (most deprived)	3.31	[1.71;	6.40]	0.001

AUC = 0.607

In imputed cases analysis (model 3), results in Table 4 show a significant association between i-IDI and stage at diagnosis, with an i-IDI-related gradient. The magnitude of OR in model 3 is higher to what was observed using IDI (model 1). These results are obtained on a sample that did not exclude non-respondent women and reduced, if not eliminated, the selection bias present in complete case analysis. In this same population, in proxy measure analysis (model 4), results using EDI instead of IDI or i-IDI shows a significant association between EDI and stage at diagnosis, with an EDI-related gradient (Table 4).

**Table 4:** Results on the full sample representing the 1644 women initially included, regardless of whether they were respondents or non-respondents. Analysis addressing the crude association (Odds ratios [95% confidence intervals]) between stage at diagnosis (advanced vs early stage (ref.)) and SEP measured by i-IDI (imputed case analysis (Model 3)) or EDI (proxy measure analysis (Model 4))

	*N* = 1644	OR	[95%CI]		*p*-value
Imputed case analysis (model 3)					
imputed Individual Deprivation Index (i-IDI)	0 (least deprived)	1			
	1	1.13	[0.85;	1.50]	0.393
	2	1.35	[0.97;	1.88]	0.073
	3 (most deprived)	2.03	[1.38;	2.99]	<0.001
AUC = 0.576					
Proxy measure analysis (model 4)					
European Deprivation Index (EDI)	quintile 1 (least deprived)	1			
	quintile 2	1.29	[0.98;	1.71]	0.073
	quintile 3	1.24	[0.93;	1.66]	0.144
	quintile 4	1.43	[1.05;	1.94]	0.024
	quintile5 (most deprived)	1.48	[1.06;	2.08]	0.022

AUC = 0.537

Regarding AUC, the model using i-IDI (model 3) seemed to produce less classification errors than models using IDI in model 1.a (p-value = 0.0418), or EDI in model 1.b (*p*-value = 0.0073) and in model 4 (*p*-value = 0.0497). In sensitivity analyses, using models adjusted for age did not change our observations and conclusions. In the analysis excluding EDI from the imputation model, it provided lower regression coefficients, OR [95% CI]: 1.10 [0.85; 1.43] (*p*-value = 0.477), 1.32 [0.95; 1.83] (p-value = 0.101), and 1.86 [1.27; 2.73] (p-value = 0.002) respectively for i-IDI 1, 2 and 3 with 0 as reference.

## Discussion

Our study illustrates how area-level SEP measures may be used to deal with missing data on individual-level SEP measures in the analysis of social inequality in health, here, the stage at diagnosis in breast cancer. We showed that complete cases analysis would lead to a selection bias that would results to an underestimation of the magnitude of the association of interest, even in case of high response rate. Rather than focusing on individual with data complete data on individual SEP, using EDI to impute missing individual SEP data provides results that are not subjected to selection bias. Compared to complete cases analysis, the increase observed in the magnitude of the OR in imputed cases analysis seems to confirm the interest to include women with missing data on social deprivation and then to use EDI for imputing it. Moreover, using EDI to impute missing individual SEP rather than using it in replacement of individual SEP provides results that are not subjected to the loss of accuracy and power deriving from the use of an ecological index to approach the individual level. Based on models AUC, we observed that using EDI in place of individual SEP measure lead to models that are likely to have poorer goodness of fit.

The main limitation of this work is to discuss the possibility of dealing with selection bias in incomplete individual-level data with an ecological measure using only one tool, i.e. the EDI. However, in a paper published in the early 2017, Bryère et al. compared the performance of the main ecological deprivation index by assessing the ecological bias by measuring the misclassification of individual SEP in seven ecological indices (Townsend index, Carstairs index, Lasbeur index, Havard index, the social (SCP) and material (MCP) components of Pampalon index, and the European Deprivation index (EDI)) used at the IRIS level [17]. They found that the aggregate indices studied were quite good “proxies” for SEP (Area Under the Curve close to 0.7), and they had similar performances. However, the indices were more efficient at measuring individual income than education or occupational category and are suitable for measuring deprivation but not affluence [17]. Despite these limitations, EDI was used in previous paper to study social inequalities in cancer risk [20], incidence [21], treatment [22, 23], characteristics [24] or outcome [25] confirming the relevance of such index. Since its development in 2012, almost forty papers using the European Deprivation Index (EDI) was published up to the first quarter of 2019. In more than half of these papers, no individual data were available, and the EDI was used as a proxy of individual-level SEP. In the others, EDI was used to characterize the contextual environment alone (12 papers) or in complement of individual-level measures of SEP (4 papers, see Additional file 1). To our knowledge, this is the first time the EDI is used to impute for missing individual SEP.

In this work we were interested in how the choice of a SEP index (IDI, EDI or i-IDI) modified the association between individual SEP and stage at diagnosis. That is why we presented crude OR in models addressing the association between SEP and stage at diagnosis. Therefore, we were not surprised by the relative smallness of the AUC because these models may miss some important intermediate and confounding variables in the relation between SEP and stage at diagnosis. Nevertheless, this is not a limitation regarding our objective of discussing the use of ecological-level SEP measure to deal with missing data when individual measures are available but uncompleted. As the measure of SEP varies between proxy measure, complete, missing and imputed cases analyses, we cannot assess directly whether the multiple imputation has reduced the influence of the women excluded for missing data in the results regarding the magnitude of the association. However, the comparison of the results from the different analyses indicates that the association between SEP and outcome is strengthened by the inclusion of the 426 women with missing data on IDI. In the missing case analysis, the strength of the association between SEP and stage at diagnosis seemed to be strong enough among these women to be observed using EDI despite the lack of power and accuracy linked to the use of aggregated data. The results confirm what was observed in Table 1 regarding the profile of the non-respondent women and show that complete case analysis would underestimate the “real” strength of the association between stage at diagnosis and SEP. Results from proxy measure analysis are not in contraction with what was expected as this model includes 426 women among whom a strong EDI-outcome gradual association was found and 1218 women among whom no EDI-outcome association was found. The magnitude of the associations between SEP and stage at diagnosis presented in Table 4 are likely to be underestimated since EDI is used as a proxy of individual level SEP as illustrated in complete case analysis. Our results advocate for choosing ecological deprivation index to impute missing data on individual SEP as it allows for working on the full sample and it yields the best goodness of fit. Moreover, sensitivity analysis excluding EDI from the imputation model provides lower regression coefficients, than those obtained in including EDI. This confirmed the interest of using ecological deprivation index to impute individual SEP to avoid underestimation of the SEP-stage at diagnosis association, especially for the most deprived.

The results from missing data analysis seems not incompatible with the assumption that missing values are generated following a missing at random (MAR) mechanism [16]. Under this assumption, the risk of having biased results using required for applying multiple imputation procedure is weak [18]. Although results showed differences in the proportion of patients living in the areas with the highest deprivation level between respondents and non-respondents, they did not show a statistically significant association between SEP assessed by EDI in quintile and the fact of being non-respondent. EDI did not strictly reflect the real individual SEP assessed by IDI, but these variables are correlated (spearman rho = 0.116 (*p* < 0.001)). In imputation models, we include stage at diagnosis as it has been shown that using the outcome for imputation of missing predictor values provided less biased regression coefficient than imputation without the outcome which provided biased and underestimated estimations [26]. Finally, our results are in line with previous study that supported the interest of multiple imputation regarding complete case analysis, in particular in the case of informative missingness [18, 27, 28]. We also highlight the possibility that may offer ecological SEP measure for assessing selection bias due to incomplete date on individual-level measures.

## Conclusion

This work illustrates the interest of using ecological measure of SEP to assess the bias that may occur in complete cases analyses but also to deal with missing data in individual SEP. When individual data are at least partially available, we argue in favour of using ecological index to assess the potential selection bias and for imputing missing individual SEP rather than using ecological index in replacement of individual SEP to avoid the ecological bias due to the use of aggregated data to infer to individual level.

## Additional file

Additional file 1: List of the papers found using EDI. (DOCX 35 kb)

## Data Availability

The datasets generated and/or analysed during the current study are not publicly available as the data belongs to the cancer registries of the Francim network but are available from the corresponding author on reasonable request.

## Abbreviations

AUC: area under the curve
EDI: European deprivation index
IDI: individual-level deprivation index
i-IDI: imputed individual-level deprivation index
OR: odds ratio
SEP: socioeconomic position
