# Supplementary material for: Using ecological socioeconomic position (SEP) measures to deal with sample bias introduced by incomplete individual-level measures: inequalities in breast cancer stage at diagnosis as an example
Source: BMC Public Health. 2019 Jul 2;19:857. doi: 10.1186/s12889-019-7220-4 (PMC6604477; doi:10.1186/s12889-019-7220-4)
Supplement: Supplementary file 1 — List of the papers found using EDI. (DOCX 35 kb) [file 12889_2019_7220_MOESM1_ESM.docx]

Supplemental file

List of the papers we found using EDI either in complement to individual SEP measures (1-4) or an environmental exposure (5-16), from a Pubmed research using “european deprivation index”[title /abstract] between 2012 and now (May 2019).

1. Albouy-Llaty M, Limousi F, Carles C, Dupuis A, Rabouan S, Migeot V. Association between Exposure to Endocrine Disruptors in Drinking Water and Preterm Birth, Taking Neighborhood Deprivation into Account: A Historic Cohort Study. Int J Environ Res Public Health. 2016;13(8).

2. Bryere J, Menvielle G, Dejardin O, Launay L, Molinie F, Stucker I, et al. Neighborhood deprivation and risk of head and neck cancer: A multilevel analysis from France. Oral oncology. 2017;71:144-9.

3. Ouidir M, Lepeule J, Siroux V, Malherbe L, Meleux F, Riviere E, et al. Is atmospheric pollution exposure during pregnancy associated with individual and contextual characteristics? A nationwide study in France. J Epidemiol Community Health. 2017;71(10):1026-36.

4. Ouedraogo S, Dabakuyo-Yonli TS, Roussot A, Pornet C, Sarlin N, Lunaud P, et al. European transnational ecological deprivation index and participation in population-based breast cancer screening programmes in France. Prev Med. 2014;63:103-8.

5. Belot A, Remontet L, Rachet B, Dejardin O, Charvat H, Bara S, et al. Describing the association between socioeconomic inequalities and cancer survival: methodological guidelines and illustration with population-based data. Clinical epidemiology. 2018;10:561-73.

6. Bryere J, Dejardin O, Bouvier V, Colonna M, Guizard AV, Troussard X, et al. Socioeconomic environment and cancer incidence: a French population-based study in Normandy. BMC Cancer. 2014;14:87.

7. Bryere J, Dejardin O, Launay L, Colonna M, Grosclaude P, Launoy G. Socioeconomic status and site-specific cancer incidence, a Bayesian approach in a French Cancer Registries Network study. Eur J Cancer Prev. 2018;27(4):391-8.

8. Dialla PO, Arveux P, Ouedraogo S, Pornet C, Bertaut A, Roignot P, et al. Age-related socio-economic and geographic disparities in breast cancer stage at diagnosis: a population-based study. Eur J Public Health. 2015;25(6):966-72.

9. Fournel I, Bourredjem A, Sauleau EA, Cottet V, Dejardin O, Bouvier AM, et al. Small-area geographic and socioeconomic inequalities in colorectal tumour detection in France. Eur J Cancer Prev. 2016;25(4):269-74.

10. Michel M, Bryere J, Maravic M, Marcelli C. Knee replacement incidence and social deprivation: results from a French ecological study. Joint Bone Spine. 2019.

11. Morelli X, Rieux C, Cyrys J, Forsberg B, Slama R. Air pollution, health and social deprivation: A fine-scale risk assessment. Environ Res. 2016;147:59-70.

12. Ribeiro AI, Krainski ET, Carvalho MS, De Fatima de Pina M. The influence of socioeconomic deprivation, access to healthcare and physical environment on old-age survival in Portugal. Geospatial health. 2017;12(2):581.

13. Rochoy M, Raginel T, Favre J, Soueres E, Messaadi N, Deken V, et al. Factors associated with the achievement of cervical smears by general practitioners. BMC Res Notes. 2017;10(1):723.

14. Rollet Q, Bouvier V, Launay L, De Mil R, Launoy G, Dejardin O, et al. No effect of comorbidities on the association between social deprivation and geographical access to the reference care center in the management of colon cancer. Digestive and liver disease : official journal of the Italian Society of Gastroenterology and the Italian Association for the Study of the Liver. 2018;50(3):297-304.

15. Temam S, Varraso R, Pornet C, Sanchez M, Affret A, Jacquemin B, et al. Ability of ecological deprivation indices to measure social inequalities in a French cohort. BMC Public Health. 2017;17(1):956.

16. Tron L, Belot A, Fauvernier M, Remontet L, Bossard N, Launay L, et al. Socioeconomic environment and disparities in cancer survival for 19 solid tumor sites: An analysis of the French Network of Cancer Registries (FRANCIM) data. Int J Cancer. 2019;144(6):1262-74.
